# Are Colon and Rectal Cancer Two Different Tumor Entities? A Proposal to Abandon the Term Colorectal Cancer

**DOI:** 10.3390/ijms19092577

**Published:** 2018-08-30

**Authors:** Stephan Paschke, Sakhavat Jafarov, Ludger Staib, Ernst-Dietrich Kreuser, Catharina Maulbecker-Armstrong, Marc Roitman, Torbjörn Holm, Curtis C. Harris, Karl-Heinrich Link, Marko Kornmann

**Affiliations:** 1 Department of General and Visceral Surgery, University Hospital Ulm, 89081 Ulm, Germany; stephan.paschke@uniklinik-ulm.de; 2 Research Group Oncology of Gastrointestinal Tumors (FOGT), University of Ulm, 89081 Ulm, Germany; l.staib@klinikum-esslingen.de (L.S.); ernst-d.kreuser@vodafone.de (E.-D.K.); k-h.link@asklepios.com (K.-H.L.); 3 Surgical Clinic, University Hospital Duesseldorf, 40225 Duesseldorf, Germany; saxavet.jafarov@yahoo.de; 4 Department of General and Visceral Surgery, Hospital Esslingen, 73730 Esslingen, Germany; 5 Department of Hematology and Oncology, KH Barmherzige Brueder, 93049 Regensburg, Germany; 6 Department of Disease Prevention, Hessian Ministry for Social Affairs, Health, and Integration, Germany and Technical High School Giessen, 35390 Wiesbaden, Germany; maulbecker@hotmail.com; 7 Surgical Center and Asklepios Tumor Center, Asklepios Paulinen Klinik, 65197 Wiesbaden, Germany; m.roitman@asklepios.com; 8 Department Molecular Medicine, Coloproctology, Centre of Surgical Gastroenterology, Karolinska University Hospital, SE-171 76 Stockholm, Sweden; torbjorn.holm@sll.se; 9 Laboratory of Human Carcinogenesis, Center for Cancer Research, National Cancer Institute, Bethesda, MD 20892, USA; harrisc@mail.nih.gov; 10 Hessian and German Cancer Societies, 14057 Berlin, Germany

**Keywords:** colorectal cancer, colon cancer, rectal cancer

## Abstract

Colon cancer (CC) and rectal cancer (RC) are synonymously called colorectal cancer (CRC). Based on our experience in basic and clinical research as well as routine work in the field, the term CRC should be abandoned. We analyzed the available data from the literature and results from our multicenter Research Group Oncology of Gastrointestinal Tumors termed FOGT to confirm or reject this hypothesis. Anatomically, the risk of developing RC is four times higher than CC, while physical activity helps to prevent CC but not RC. Obvious differences exist in molecular carcinogenesis, pathology, surgical topography and procedures, and multimodal treatment. Therefore, we conclude that CC is not the same as RC. The term “CRC” should no longer be used as a single entity in basic and clinical research as well as other areas of classification.

## 1. Introduction

Colon cancer (CC) and rectal cancer (RC) are regarded in all fields of research and clinical practice a single tumor entity termed colorectal cancer (CRC). This is based on the assumption that CC and RC develop in the large bowel, which is thought to be a single organ. The border to the oral edge is the ileocecal valve of the small bowl and the border to the anal edge the dentate line to the anal canal, sphincter ani, and skin. The term CRC has been based on the similar anatomical structure consisting of mucosa, muscular layer, and in part serosa, their function of stool concentration, fluid resorption, stool transportation and excretion, and similar histology. Our groups have been working for decades on CC and RC in basic, translational, and clinical research. This included German national projects to structure and improve treatment of CC and RC patients (Research Group Oncology of Gastrointestinal Tumors (FOGT), participation in S3 guide line committees, structural commissions of the German cancer society to establish large bowel centers, and projects for disease prevention for the Hessian and German Cancer Societies. In addition, we analyzed the literature about landmark characteristics to find out if significant similarities between CC and RC exist justifying the term CRC or, if not, allowing us to reject it. The results of our two large multicenter multimodal treatment studies about adjuvant chemotherapy of CC (FOGT1) [1] and RC (FOGT2) [2] were included in the analytic set up. We strongly believe that there is now enough evidence to end the discussion that has been smoldering for decades and to divide CRC into CC and RC. Rejection of the term CRC would divide CC and RC as self-standing tumor entities.

## 2. Results

### 2.1. Anatomy and Topography

Anatomically and topographically the rectum is defined as large bowel up to the edge of 16 cm from the anocutaneous (AC) line. The lower third reaches up to 6 cm from the AC line, the middle third ranges from 6–12 cm and the upper third from 12–16 cm from the AC line. The upper third has an intraperitoneal position, while the lower two thirds are located extraperitoneally in the small pelvis. The upper edge of the rectum may also be defined by the confluens of the three colon tenias to a single rectal tenia. The topography of the upper third varies between males and females. The two lower thirds have a sophisticated topography concerning the mesorectal structures, fascias, nerval and vascular anatomy, and position adjacent to the ventrally located sexual organs, pelvic vessels, and nerve structures [3,4,5,6]. The venous blood from the lower two rectal thirds is drained via the internal iliac veins and the inferior caval vein into the lungs and from the upper third via the inferior mesenteric vein into the liver. The venous outflow of the colon is merely drained into the liver via the inferior (colon sigmoideum and colon descendens) and superior (transverse colon and colon ascendens) mesenteric veins. The arterial blood supply of the colon descendens, sigmoid colon, and the upper rectal third originates from the inferior mesenteric artery, while the rest of the colon is supplied by the superior mesenteric artery. The lower two rectal thirds receive their arterial blood supply via the internal iliac arteries [6,7,8,9,10]. The lymphatic drainages of the rectum are led in parallel to the inferior mesenteric vein (upper third) or along the pararectal, internal iliac lymph streets (middle third), or along the inferior rectal artery (lower third) [7,8,10]. The innervation of the rectum is supplied by the superior and inferior hypogastric plexus (superior plexus = N. sympaticus; inferior plexus = N. sympaticus and N. parasympaticus) as well as for the pelvic organ function [8,9,11,12]. The nerve supply of the colon runs along the arterial vascular supplies as described above. Based on the anatomy and topography especially of the lower two thirds of the rectum, RC is much more demanding for the surgeon than CC. Special skills are required for surgical procedures and the curative limits in T4 stages of both entities. Surgery of RC with the aim of sphincter preservation is even more demanding.

In summary, the topographic position and the anatomy of the rectum and its function impose more perception on the patient than the colon. Surgical treatment of RC imposes more challenges and risks for malfunction and irreversible damage concerning continence and lesions of surrounding structures resulting in major bleeding or malfunctioning pelvic organs on the surgeon.

### 2.2. Epidemiology

CCs and RCs are usually epidemiologically registered as CRCs. The incidence of CRC in Europe is higher than in Africa or Asia but lower than in the US [13,14]. It is associated with nutritional habits concerning fat and meat consumption [15]. The sex distribution for CRC favors male (53%) vs. female (47%). The risk to develop RC is 1.5 higher in males than in females, while females are predominant in developing cancers in the proximal colon (1.2:1). In the last four decades, there was a “shift to the right” with increasing incidences of cancers in the right hemicolon. Currently, 15–35% of the cancers are located in the rectum and 25% in the right hemicolon [16,17,18,19,20]. Out of 129,700 newly registered CRCs, 93,090 (72%) were diagnosed in the colon and 39,610 (28%) in the rectum according to the statistics of the American Cancer Society in 2015 [21], resulting in a proportion of 2.5:1 (CC:RC). This may suggest that the carcinogenic risk in the colon is higher than in the rectum. There is a shift to the right, suggesting that the incidence of CC is increasing while that of RC and left sided CC is decreasing.

### 2.3. Carcinogenic Risk

In contrast to the before described epidemiologic data, in our opinion, at least the carcinogenic risk of the rectal mucosa to develop cancer by far exceeds that of the colon mucosa inasmuch as the area at risk in the colon is definitely larger than that of the rectum. The total area at risk can be calculated from the length of the colon (~150 cm) and rectum (~16 cm). The incidence of CC per cm in the US in 2015 [21] is 621 cm^−1^ (93,090/150 = 620.6) and that of RC 2,479 cm^−1^ (39,610/16 = 2,478.6) resulting in a relative risk per cm of at least 1:4 for colon and rectum. In other words, the rectal mucosa has an at least four times higher risk for malignant transformation than the colon mucosa. This may either depend on various susceptibilities to carcinogens or to different carcinogenic processes in the colon and rectum.

### 2.4. Formal Carcinogenesis

In formal carcinogenesis 85–90% of the cancers arise from low grade or high grade intraepithelial neoplasias (LGIN, HGIN) mainly in form of adenomas [22]. These are classified as tubular (75%), villous (10%), or mixed (15%) with a malignant transformation risk of 5% for tubular adenomas, 19% for mixed, and 39% for villous [11,23]. Most of the malignant tumors are mucinous or non-mucinous adenocarcinomas, others have signet ring, anaplastic, or squamous cell differentiation with worse prognosis [24]. Low risk cancers (L0) are well or moderately differentiated (G1, G2), high risk cancers (L1) are poorly differentiated or undifferentiated (G3, G4) [3,22,25,26].

### 2.5. Macroscopy and Histopathology

According to their macroscopic form of growth, four forms of CC are distinguished: bowel shaped and ulcerating (55–60%), polyp-cauliflower (25%), flat (15–20%), and diffuse infiltrating (1%). Exophytic growth is predominant in the proximal colon, while growth is endophytic ring–shaped in the distal colon [25,27]. RC may grow exophytically, endophytically with ulcerations and intramural expansion, or diffuse infiltrating with linitis plastica [8]. CC and RC therefore may have different macroscopic appearances.

**Figure 1:**
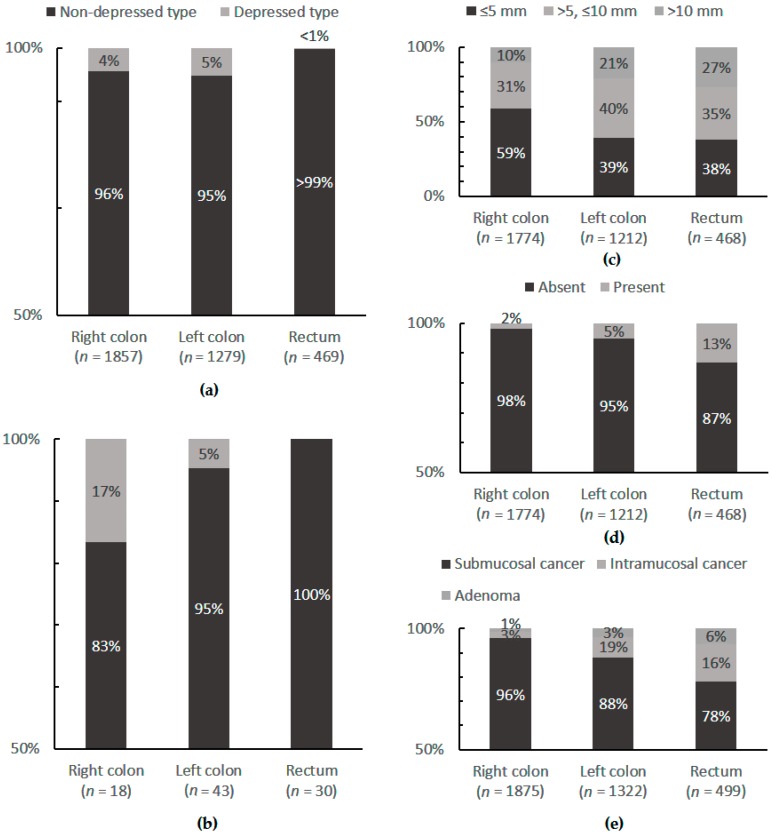
Comparison of the incidences of depressed and non-depressed types of neoplastic lesions in the rectum, the left colon, and the right colon. (**a**) Mucosal lesions; (**b**) submucosal cancers. A significant difference in the macroscopic type was noted between the rectum and the colon (*p* < 0.001). The incidence of depressed submucosal cancers in the right colon was significantly higher than that in the rectum (*p* = 0.0472); (**c**) Relationship between the location and the size of non-depressed mucosal lesions; (**d**) Relationship between the location and the incidence of villous components in non-depressed mucosal lesions; (**e**) Locations of mucosal lesions and submucosal cancers. (Adapted from Konishi et al. 1999 [28].)

In early cancer, histopathologically mucosal lesions of the polypoid, non-depressed type are more frequently found in the colon than in the rectum (right hemicolon 51%, left hemicolon 35%, rectum 14%), while submucosal lesions more frequently occur in the rectum (Figure 1a). Mucosal lesions with villous components were found more frequently in the rectum (13%) than in the left (5%) or right (2%) hemicolon [28]. The absolute values for frequency of mucosal and submucosal lesions of the depressed type are higher in the rectum than in the right colon (Figure 1b) [28]. The Japanese authors describing this phenomenon, suggest that different carcinogenic mechanisms are the reason for this differing histopathologic appearance of CC and RC [28].

### 2.6. Hereditary Syndromes and Molecular Carcinogenesis

The difference in the incidences of hereditary syndromes involved in the development of CC and RC implicates as well that molecular carcinogenesis of CC seems to be different from that of RC. Hereditary non-polyposis colorectal cancer (HNPCC) manifests predominantly in the colon, especially proximal colon, while the familial adenomatous polyposis coli syndrome (FAP) is predominantly causing cancer in the distal colon and rectum, but can also occur in the rest of the colon [29,30]. Several characteristic differences between HNPCC and FAP are shown in Table 1.

Macroscopically the APC type shows a polypoid growth pattern, while that of the MSI type is flat (HNPCC). MSI types more frequently occur in the right than in the left colon (44% vs. 25%), while polypoid cancers are more frequent in the left (59%) than in the right (40%) colon [31]. The flat growing early precursors of cancers are significantly more difficult to detect than the polypoid growing [31,32,33].

CC and RC from a molecular biological point of view may be regarded as MSI or APC type. MSI types are more frequent in the proximal colon and flat, while APC types are polypoid. CC and RC differ in their chromosomal and molecular profiles as well as in enzyme expressions. However, there is no clear cut boundary between rectum and descending colon [34].

Analyzing molecular carcinogenic alterations of CC and RC differences in molecular profiles and enzyme expression patterns become evident (Table 2). As mentioned above MSI is more frequently detected in proximal CC than in RC [35,36,37,38,39], which is also the case in HNPCC patients [30,40,41,42]. Proximal CC more frequently shows mutations (V600E) in the Serin/Threonin-Kinase-BRAF [37,43,44], or an expression of the CPG-island methylator phenotype (CIMP) [45,46,47], high gene expression of HOX [46] and CDX2 [48], increased mutations of KRAS [49], and higher activity of the MAPK signal transduction pathways [50]. In distal CC and RC the following changes and molecular characteristics are more frequent compared to proximal CC: Positivity of chromosome instability (CIN) [45], stability of microsatellites (MSS) [29,30,51], which is also the case for FAP [52]. EGFR or HER2 gene amplificationes are found [53] as well as p53 mutations [49,54]. Wnt signal pathways in carcinogenesis are activated more often as well [40,55,56,57]. The importance of p53 for the carcinogenesis of CC and RC has been extensively described [58]. The entities also differ in protein expression levels, which are higher for Cyclin D3 and cMyc in CC, and for Cyclin D1, Cyclin E and nuclear beta-Catenin in RC [49]. High thymidylate synthase (TS) expression correlates with better survival in the spontaneous course [54,59,60] or after adjuvant chemotherapy [61,62,63,64] in CC. Vice versa in RC high TS is either associated with poor survival [65,66,67] or is meaningless [68].

In summary the hereditary cancer syndromes HNPCC (2–7% of all CRCs) and FAP (1% of all CRCs) are differing in their molecular chromosomal changes [34]. FAP has an APC gene mutation (APC type; 60% of all CRCs), while in HNPCC the germ chromosomes are mutated in their DNA information for MMR genes (MLH1, MSH2, MSH6, PMS1, and PMS2) leading to MSI (MSI type) [40,41,69]. CCs and RCs may be categorized according to the features of the APC type (about 2/3 of CC + RC) and the MSI type [26,70,71].

The results of an international consortium which analysed molecular, enzymatic, and immunogenic characteristics and microscopic growth patterns including angiogenesis were innovative for the classification of CRC. With their data collection the CRC Subtyping Consortium (CRCSC) defined four robust consensus molecular subtypes CMS 1–4 [72]. Most interestingly, CMS1 tumors were frequently diagnosed in females with right sided lesions and presented with higher histopathological grade. CMS2 tumors were mainly left sided. CMS3 showed mutated ras and metabolic dysregulation, low frequency of CIMP and gene copy alterations, and a mixed MSI status. CMS4 tumors tended to be diagnosed at more advanced stages (UICC III and IV) and displayed worse overall and relapse free survival (in the multimodal PETACCC-3 trial involving adjuvant CT in CC UICC III). More recent results point out that CMS2 and 3 subtypes are relatively more immune suppressed compared to CMS1 and 4, opening up new possible treatment options including PD-1 receptor blockage [73]. After relapse (and treatment of relapse), survival was superior in CMS2 patients and very poor in the CMS1 population [72]. The differences of CC and RC focusing on molecular changes and differences in protein expression levels of our literature analysis, many of which are included in the CMS subtyping, are summarized in Table 2. These data reveal that the proximal and distal colon and the rectum show significant differences.

### 2.7. Primary Preventive Measures

A difference in carcinogenic principles could also be the reason for the different effectiveness of primary preventive measures through physical activity on the development of CC and RC. Various groups recently reported that physical activity at higher levels could reduce the incidence and/or risk of CC by up to 40% [74,75,76,77], but this was without effect in RC [75,78,79,80]. In the Cancer Prevention Study II Nutrition Cohort with 70,403 men and 80,771 women the risk of CC was reduced by 16% (RR 0.84, 95% CI 0.59–1.20) in participants who actively exercised (79% of all study participants), while this was not observed in RC [79]. The results of various preventive studies with sportive activities, BMI, reduced energy intake, or medical intervention with COX-2 inhibitors or aspirin are summarized in Table 3. In summary, physical activity may reduce the risk of CC, but not of RC.

### 2.8. Clinical Symptoms and Examinations

CC patients most often have other clinical symptoms than RC patients. CC may be detected due to anemia, abdominal pain, meteorism, paradoxical diarrhea, and fatigue. RC may become clinically evident by overt blood admixtures to the stool, pencil-stools, incontinence and pain while sitting. Complete Colonoscopy with tumor biopsies, abdominal ultrasound or abdominal CT-scan, X-ray or computed tomography of the lungs, and serum-CEA determination are necessary for both CC and RC staging. An MR of the pelvis evaluated by a specialized radiologist is necessary in RC. Hereditary syndromes mentioned above must be excluded.

### 2.9. Surgery

Today, the principles of mesorectal [4,87,88] or mesocolonic excision [89] together with resection of the regional lymphatic draining mesenteric areas/parts of the tumor bearing large bowel sections are standard and recommended by many national guidelines. Overall, surgery of CC is less demanding than that of RC as already mentioned. The surgical principles follow the tumor location and involve right/left hemicolectomies, and transversal or sigmoid/rectosigmoid resection [90]. RC surgery is methodically more complex, and the surgical demands increase with the depth of the aboral tumor position and closer to the sphincter muscle [3,8,91]. Tumors localized in the lower two thirds of the rectum which receive a sphinkter-preserving procedure usually also need a move to improve continence in form of a pouch procedure [3].

Consequently, RC surgery is associated with higher morbidity/mortality and with higher local relapse rates than CC surgery [91,92,93,94]. Specific postoperative morbidity in a large prospective observational study by the “Institute for Quality Assurance, Magdeburg” involving 3756 Patients was 21.8% in resected CC patients and 29.1% in resected RC patients. Anastomotic insufficiency was registered after 3% of CC and 9.5% of RC resections [95]. RC surgery may be associated with severe functional and physical disadvantages summarized as low anterior resection syndrome (LARS) [39,96]. Functional dysturbances after RC surgery are much more frequent compared to CC surgery. A comparison of quality of life after CC versus RC surgery showed that overall there is no difference, however functional scores and items with regard to defecation are more frequently unsatisfying for patients after RC surgery [39,96].

In spite of the fact that large phase III trials came to identical results when comparing open vs. laparoscopic surgery [97,98,99,100,101,102,103], laparoscopic surgical resection of CC is more frequently practiced than of RC. It must be pointed out that the conversion rate in laparoscopic CC is lower than in RC. Conversion ameliorates the surgical treatment results [104,105,106]. The results in mere laparoscopic vs. conversion vs. mere open surgery in 7189 patients with RC reported by Ptok et al. [104] from their multicenter observational study are shown in Table 4.

The laparoscopic learning curve in RC is higher than in CC and laparoscopic RC surgery should be performed by specialists only [106,107]. It must be kept in mind that due to the intraperitoneal vs. extraperitoneal location of CC vs. RC multivisceral resections in case of T4 tumors are more frequent in CC (e.g., 12.4%) than in RC (e.g., 6.9%) [92,93]. Since CC may turn symptomatic at a later stage emergency surgery as well is more frequent in CC (e.g., 20%) than in RC (e.g., 6%) [93,94]. The long-term results after curative surgery followed by adjuvant multimodal therapy for CC (FOGT-1) [1] and RC (FOGT-2) [2] are shown in Table 5. The seven-year-OAS-rates in RC stages UICC II and III are consistently lower than those in CC. The lower survival rates in the spontaneous course without multimodal therapy of RC vs. CC have also been demonstrated in our large data base of patients who had resection for CC or RC without adjuvant CT or neoadjuvant/adjuvant RCT + CT [108].

In summary, emergency surgery has to be performed more frequently in CC than in RC. For elective cases, laparoscopic surgery for CC requires less expertise than for RC. Morbidity after RC surgery is higher than after CC surgery. Local relapses occur more frequently after RC surgery than after CC surgery. The disease pattern after curative surgery of CC is different from that of RC in the spontaneous course and after multimodal treatment. Long-term survival rates in CC are higher than in RC without and with multimodal therapy.

### 2.10. Biology

From the biological point of view CC and RC have different characteristics as well. CC and RC differ in their relapse patterns in the spontaneous course after curative surgery, especially with regard to local relapses (Table 6). In addition, right sided CCs more frequently develop peritoneal dissemination, while left sided more frequently develop liver and lung metastases [19]. The metastatic and recurrence pattern of CC and RC after adjuvant treatment are also different. Despite the fact that in our two adjuvant phase III trials of CC (FOGT-1) and RC (FOGT-2) a similar adjuvant chemotherapy protocol had been applied and the total recurrence rate was 36% in both trials [1,2] the pattern of recurrence was different. Metastatic target sites in CC were the liver (20.8%), peritoneum (8.9%) and lung (7.3%), while in RC the pattern was liver (19.6%), lung (12.7%), and peritoneum (4.0%) [10,109]. In RC bone marrow and other locations are more often targets of recurrence (2.1%) [109], while in CC this is an extremely rare event [110]. In summary, liver is equivalently targeted by both entities. The peritoneum is more often affected from CC, while the lung almost twice as much host for RC metastases compared to CC (Table 6).

### 2.11 Multimodal Treatment

When comparing the relapse patterns in our FOGT-1 and -2 trials, which contained similar systemic adjuvant chemotherapy principles in the three trial arms (5-FU vs. 5-FU+FA vs. 5-FU+IFN alpha), lung metastases occurred more frequently in RC (12.7%) than in CC (7.3%) after multimodal treatment [1,2,109]. Peritoneal cancer developed more frequently in CC (8.9%) than in RC (4.0%) [109]. Local relapses in the primary tumor region more frequently occurred in RC with total events (isolated and combined with distant metastases) of 12.6% in RC with prescribed surgical techniques of PME/TME and other standard procedures depending on the tumor location followed by postoperative RCT + CT compared to 8.4% in CC with prescribed surgical technique and postoperative CT. Most interestingly, adjuvant chemotherapy (CT) with 5-FU + FA increased 5-year survival of CC significantly by 12 points of percentage compared to 5-FU alone, while there was absolutely no effect of addition of FA to 5-FU in RC [1,2,109]. The relative insufficiency of systemic adjuvant therapy in RC has been addressed by others as well [113,114,115]. Intensive adjuvant chemotherapy in RC may even be harmful to older patients. We compared the effects of different treatment regimens of CC and RC in older (>70 years) and younger (≤70 years) patients. In CC (FOGT-1), survival was significantly increased by 5-FU + FA at the same level for older and younger patients [116]. Very unexpected and dissimilar to CC survival of older RC patients receiving 5-FU+FA was lower compared to 5-FU alone (5Y-OAS 45.7% vs. 63.4%; unpublished observation). In the whole study population survival did not differ significantly [2]. The relapse and progression patterns of the CC (FOGT-1) and RC (FOGT-2) trials are summarized shown in Table 7.

More recent studies demonstrated that in nodal positive CC adjuvant chemotherapy with FOLFOX4 has improved the outcome compared to 5-FU + FA (six-year overall survival 72.9% vs. 68.7%) [117]. This result basically was confirmed by the adjuvant trial NSABP-C-07 [118] and others, so that adjuvant FOLFOX now is standard for UICC III patients ≤70 years in Germany [3]. FOLFOX did improve response and local relapse rates in one RCT + CT trial [119], but not in three other trials with a similar aim/design [120,121,122], implicating that there is no convincing evidence on the highest level A (summarizing the results of two or more phase III trials with identical structures/aims) that a modulation of 5-FU or other fluoropyrimidines improves the efficacy of multimodal systemic therapy compared to “simple” RCT consisting of a fluoropyrimidine alone as a radiosensitizer during RCT and as CT thereafter. This may be due to the fact that RC is more resistant to CT than CC at least in the multimodal neoadjuvant and adjuvant setting. On the other hand, multimodal radiotherapy focused treatment consisting of RT + 5-FU (± others) or RT alone [123,124] are well established with proven effects in neoadjuvant therapy of RC, but not in CC. Neoadjuvant RCT in RC may lead to response with even complete remissions, eventually allowing to avoid surgery in high risk patients and with the possibility to resect for cure with shorter distal resection margins [32]. This cannot be achieved by neoadjuvant CT or RCT in CC.

CC and RC treatment has been improved by multimodal therapy. In CC modern chemotherapy (FOLFOX) improved the results of initial therapy with 5-FU or 5-FU + FA. In RC RCT improved the local relapse rates and survival. Neoadjuvant RT or RCT further reduced local relapse rates but did not improve survival compared to high-quality surgery alone. Modulation of FU with FA or even more aggressive chemotherapy protocols, such as FOLFOX, did not improve survival in RC. Thus, RC seems to be more resistant to systemic chemotherapy than CC. In older (>70 years) CC patients adjuvant chemotherapy generates a similar benefit as in the younger (≤70 years) patients, which was not the case in RC. There is also some evidence suggesting that metastastases from RC are less responsive to chemotherapy than CC. The chemosensitivity of metastases is dependent of the expression pattern of therapy targets. The expression pattern of these targets may vary for various metastastic sites [125,126,127,128]. Therefore, chemosensitity may depend on the location of the primary tumor as well [109].

### 2.12. Prediction of Response to Multimodal Treatment

At the beginning of multimodal therapy all substages of UICC II and III CC and UICC I to III RC received adjuvant CT and RT/RCT +/− CT, respectively. Since only 10–15% of the treated patients had a benefit as they either would have been cured by surgery alone as well or relapse despite adjuvant treatment [129] many efforts were put into individualization of multimodal therapy looking at molecular factors in tumor tissue for adjuvant treatment as well as palliative therapy [130].

Thymidylate synthase (TS) is a key target of fluropyrimidines and dihydropyrimidine dehydrogenase (DPD) is an enzyme involved in fluoropyrimidine recycling [131]. Expression levels were correlated to the benefit of multimodal therapy of CC and RC [62,130,132,133,134], but also to the spontaneous course of the diseases [65,64]. CRC patients with low intratumoral TS had a significantly longer survival than those with high TS [64]. After multimodal therapy the contrary was shown [62]. CRCpatients with high intratumoral TS had a longer survival than those with low TS. Low DPD in this analysis correlated to longer survival [62]. When looking at the correlation of TS expression in CC with adjuvant chemotherapy, Donada found the same correlation between high TS and longer survival in UICC II [63] similar to our analysis [62]. In a metanalysis involving 3497 CRC patients, Popat and colleagues [135] described that patients with low TS had a higher overall all survival than those with low TS. Obviously, the results when analyzing CC and RC together in the same study were conflicting. Enzyme expressions potentially relevant for the spontaneous course and/or the benefit of multimodal treatment were also separately analyzed in CC and RC. In the spontaneous course of RC low intratumoral TS correlated to a better outcome than high TS [34,65]. High TS also was a negative prognostic factor in RC patients receiving neoadjuvant RCT [66,67]. In a further analysis, the findings of Liersch et al. [67] could not be confirmed unanimously by the same group [68].

In contrast to TS and DPD, the MSI status together with the BRAF mutational status, however, meanwhile are recognized to select CC patients for adjuvant chemotherapy. MSI UICC II tumors and MSI BRAFwt CC and RC tumors have an excellent prognosis in the spontaneous course and do not need adjuvant chemotherapy [136,137]. Reimers and colleagues tried to generate a cocktail of prognostic factors for CRC (including MSI, TP53, KRAS (codons 12, 13), BRAF, EGFR, M30, Ki67, 18q LOH, CIMP, CIN, PIK3CA, TS, DPD etc.) which allowed an approach but no secure selection for individualizing multimodal therapy in CC or RC [39].

Sentinel LN (SNL) examination as a potential individual selective factor for multimodal therapy has also been tested in CC and RC. SNLs were more frequently identifiable in CC (99.1%) than in RC (91.5%) [138]. The SNL determination in CC is more sensitive for extra sentinel LN metastases in CC (75%) than in RC (36%) [139].

## 3. Discussion

Up to now, CC and RC synonymously are summarized together as colorectal cancer (CRC). The term CRC is used in research and clinical terminology. Basic research on causal and formal carcinogenesis usually focuses on cellular, molecular, and enzymatic changes independent of their origin in the large bowel. There still are multimodal trials around involving both CC (receiving adjuvant chemotherapy) and RC (receiving additional RCT or RT) and reporting results with disease free and overall survival for the whole study population of CRC.

In surgical oncology, the awareness has been accumulated since decades that RC is a different disease due to its topography, surgical challenge, complications, relapse pattern including local cure rates, and the necessity for sphincter preservation. With the acquisition of the knowledge from other fields, there is now increasing evidence that CC and RC are different diseases from many points of view. We examined many surrogate parameters to accept or reject this hypothesis regarding the literature and the results of our own [1,2].

In terms of epidemiology CRC has varying incidences, when continents or civilizations are compared. The male:female ratio in US statistics for incidences 2006–2010 was 1.3:1 in all CRC, but 1.6:1 in RC [20]. The location during the decades shifted from the left colon and rectum to the right colon [17,139]. Meanwhile, patients with cancer in the right colon are older and more frequently females than males [19]. The most frequent locations are the right hemicolon (48%) and the rectum (28%) [20]. Up to now, there are no exact data to show whether the proportion CC:RC shows a difference in the various continents analyzed. In Western countries, two thirds of CRC are located in the colon and one third in the rectum [3,14,20,140]. This implicates that the colon is more susceptible to develop cancer than the rectum. However, in our opinion, this is not the case with regard to the length of each organ. When setting the 2015 US incidence in relation to the length of each organ at risk, the rectum mucosa was four times more prone to malignant transformation than the colon mucosa.

In Western countries, from a macroscopic and histological point of view, colon cancers may have different growth patterns than rectal cancers [13,14,140]. The appearance of flat lesions (depressed type) is more frequent in the colon than in the rectum [28] and more difficult to detect as early lesions, while the polypoid non-depressed types with villous components (easier to detect) were more frequent in the rectum [28]. The authors contributed their observation to possible differences in carcinogenesis of the colon and the rectum [28]. When looking at the formal carcinogenesis, one first has to begin analyzing differences in the most frequent autosomal dominant inheritable CRC, namely HNPCC and FAP. HNPCC preferably is located in the right hemicolon. For FAP cancers, there is a tendency to left hemicolon and toward the rectum. Both entities differ substantially in their abnormalities on the chromosomal/DNA-mutational and enzymatic levels. FAP is an obligate precancerous disease. HNPCC may still be regarded as facultative, but has an expression rate of 50–70% [26]. The basis for FAP is an inherited mutation of the *APC* gene (initiation) with the consequence of several carcinogenic steps (promotion). About 60–70% of sporadic CC and RC have the same “APC type” formal carcinogenic pathway [26,141,142,143,144]. In HNPCC, a mutation of a gene of the mismatch repair family (MMR) is the germ defect responsible for a sequence of molecular changes, which eventually lead to CC (Lynch-Syndrome I) and rarely to RC. In addition, extracolic adenocarcinomas have been described (Lynch-Syndrome II) [40,41,69,143,145,146]. The defect responsible for HNPCC type of cancer is detected pathologically in the tumor tissue as microsatellite instable (MSI) in the inheritable syndromes but also in sporadic cancers, which are then classified as MSI type CC or RC.

The male to female proportions for tumor location (e.g., males get more RC than females, and females more proximal CC than males), the preferred locations either in the proximal colon for HNPCC and MSI type non-inheritable cancer or in the distal colon and rectum for the APC type, and our hypothesis to carcinogenic susceptibility all support the hypothesis that CC and RC are different tumor entities in terms of carcinogenic processes. When various alterations on the chromosomal, gene, or protein levels were analyzed in the tumor tissue marked differences between the “proximal colon” and the “rectum” appeared. The possibility to prevent CC by (high) physical activity, but not RC indirectly supports our hypothesis that major carcinogenetic processes in CC are dissimilar from RC.

Very interesting and new findings concerning the classification of CRC were generated by the CRCSC Subtyping Consortium in 2015 [72]. The group subclassified CRC from 4151 samples/patients according to various features from the molecular up to the histopathologic and immunogenic levels including 27 unique subtype labels into four distinct consensus molecular subtypes (CMS1-4) and an additional mixed group using a very heterogeneous tumor population from CC and RC patients. Patients were included with or without surgery, with or without multimodal therapy, with a variety of multimodal treatments. The group applied various analytical methods and very sophisticated biometrics. The CMS groups had various distinct biological properties. Most interestingly, two of the groups were associated to embryologically different parts of the colon. CMS1 to right sided lesions, and CMS2 mainly to the left sided lesions [72]. The tumor tissue data were supplied by six different working groups who either had their data from CRC or CC samples. There was no distinct differentiation between CC and RC [72]. In spite the fact that no separate views seem to have been shed on CC primary tumors as a whole vs. RC primary tumors, and that 858 samples were excluded, the new system implies that large bowel cancers seem to have significantly different characteristics. CRCSC proposes a new taxonomy of CRC reflecting significant biological differences in the gene expression-based molecular subtypes [147,148]. We think that this demand for a change in looking at CRC with a very complicated classification system is generalizing too early and is mainly based on a molecular primed classification view.

As mentioned above, CC and RC are different clinically. Surgery of RC is much more challenging than surgery of CC. However, due to the different timing of symptoms emergency surgery in CC by far exceeds the emergent indication for RC. Diagnostic procedures in RC are partly different from CC as RC requires an MRI judged by a radiologic specialist. The surgical oncological principles for CC and RC are comparable including mesocolic and mesorectal excision with removal of the draining lymphatic tissue. However, in RC surgery, more than seven different techniques are applied depending on the heights of the lower tumor edge and the size of the tumor with staging and grading. RC surgery requires more expertise from the surgeon in open as well as in laparoscopic surgery. On the other hand, CC surgery might require increased challenges in case of T4 tumors requiring multiorgan resections. Morbidity is higher after RC surgery. The long term results of RC surgery are worse than of CC surgery in the spontaneous course and after multimodal treatment at least regarding older data from the Ulm surgical school [108] and from the associated FOGT group [109]. However, this trend is changing, and prognosis of RC is meanwhile as good as that of CC if not better [1,2].

CC and RC receive a different multimodal treatment. Adjuvant CT is effective in CC, RCT + CT in RC. RC might also receive preoperative RT only [3,110,123]. Neoadjuvant CT or RCT or RT is not effective in CC. Neoadjuvant RT cannot be recommended uniquely for patients with tumors in the mid and upper rectum [149]. CT alone is not effective in RC [110]. CC and RC differ in their sensitivity to chemotherapeutic protocols as proven by our comparison from the FOGT-1 (CC) and FOGT-2 (RC) trials [1,2], which tested identical 5-FU modulations in a three arm phase III design [109]. RC seems to profit only from fluoropyrimidine mono-therapeutic protocols. 5-FU with FA after RCT might even harm older RC patients, while this combination protocol (without RCT) increased OAS significantly in young and old CC patients to the same extend [116]. Therefore, in terms of surgical oncologic treatment CC is different from RC. The metastatic pattern in case of disease progression after multimodal therapy is also different [109]. Lung metastases more frequently were detected in RC. In addition, in our competence center for the treatment of peritoneal malignant diseases isolated peritoneal carcinomatosis from CRC predominantly had arisen from CC primary tumors (unpublished observation).

Various prognostic molecular or enzymatic factors have been tested in CC, RC, and CRC in the spontaneous courses and in multimodal therapy aiming at personalized treatment. We and other groups [132,134] were the first to study the potential role of TS and DPD to individualize patient selection for adjuvant/neoadjuvant and for palliative treatment in CRC [62,150,151] and conducted the first prospective randomized trial for treatment of metastases [130] to introduce individualization to surgical oncology. Comparing the results from multimodal trials about molecular prognostic factors possibly used for treatment individualization reveal rather different results for CC and RC. Reimers et al. have suggested a cocktail of modern prognostic factors for patient selection in neoadjuvant treatment of RC [39]. Due to the lack of unanimous convincing data this approach is far from routine yet.

Treatment individualization may be possible in metastatic disease using fluropyrimide modulating protocols as well [130,150,151,152]. However, nobody so far distinguished the primary origin in metastatic CRC disease. With the aim of treatment individualization for adjuvant and palliative CT, Yamada et al. reported that in rectal cancer TS concentration in lung metastases are higher and in liver metastases lower than in the primary tumor [127]. Meanwhile, there are reports, that metastases from rectal cancer show a different response than those from colon cancer [125,126,127,128], again an argument for our hypothesis that CC is different from RC.

Currently there is no proof that RC and CC are significantly different with regard to prognostic and predictive factors to individualize multimodal or palliative therapy. Data about the impact of molecular markers for patient selection for multimodal therapy are often still conflicting. One reason might be that the majority of the older studies combined CC and RC samples as CRC for analysis. Additional aspects including modern medical treatment like growth factor receptor and immune checkpoint inhibition may also turn out to be clearly different for CC and RC. More research is necessary in prospective trials separately investigating CC and RC to identify reliable markers for each entity, and to even split CC into right-sided and left-sided CC.

## 4. Materials and Methods

For recapitulating the basic known information, we described the current anatomical/topographical definitions of the colon and the rectum, the macro- and histopathology of colon and rectal cancers in standard literature/books/actual S3 guide lines for “CRC”. Then, we analyzed actual reports (papers and abstracts) in English about epidemiology, etiology, formal and molecular carcinogenesis, hereditary syndromes, preventive possibilities, clinical symptoms, diagnostic procedures, surgical procedures, multimodal therapies, follow up, and short/long term results. We analyzed more than 2000 publications available from Pubmed, Medline etc. concerning these fields between 2000–2016 using the keywords “colorectal cancer”, “colon cancer”, “rectal cancer”, “chemosensitivity of colon and rectal cancer”, “chemotherapy”, “surgery”, “radiochemotherapy”, “randomized clinical trials”, “molecular biology”, “prognostic factors”, and others. We also took information from the German S3 Guide Lines “Colorectal Cancer” from the versions 2008 and 2013. Results from the data bases from the FOGT trials on improvement of multimodal adjuvant CT in CC (FOGT-1) [1] and adjuvant RCT in RC (FOGT-2) [2] and associated publications were used to substantiate or reject our hypothesis.

## 5. Conclusions

We collected and analyzed data on various relevant fields to question the term colorectal cancer (CRC) and to replace it by colon cancer (CC) and by rectal cancer (RC). With regard to our findings and ample experience on several of the described fields, we came to the conclusion to accept CC and RC as different tumor entities in all aspects of experimental and clinical research and to abandon the term CRC. Basic and clinical scientist and physicians should respect this change of nomenclature and concentrate to describe results separately for RC and CC, even subdivided in right and left. These actions will help in the future to improve the accuracy of results and to improve the individualized treatment of CC as well as of RC patients.

## Figures and Tables

**Table 1:** Differences between familial adenomatous polyposis coli (FAP) and hereditary non-polyposis colorectal cancer (HNPCC).

Characteristic	FAP	HNPCC
Prevalence rate	1% of all colon and rectal cancers	5% “CRC”
Phenotype	>100 polyps	Only a few polyps can be present
Genotype	APC gene mutations	Germline mutations of the DNA MMR genes
Age of onset	In most cases from 20 to 25 years	On average from year 44 onwards
Localization	Left colon, rectum; associated disease locations: bones, eyes, duodenum	Right colon; associated disease locations: endometrium and also (considerably rarer) stomach, ovaries, pancreas, ureter, renal pelvis, cystic ducts
Transformation to colon and/or rectum cancer	100% to colon and/or rectum cancer	50–70% to colon cancer

**Table 2:** Differences in carcinogenesis, molecular genetic profile, histopathology, and biology of sporadic colon cancer (CC) compared with rectal cancer (RC).

Mutation/Expression	Proximal CC	Distal CC and RC	Author (s)
Chromosome instability (CIN)	NO	YES	Ogino et al. 2008 [45]; Smith et al. 1993 [52]
Microsatellite instability (MSI)	YES	NO	Jass et al. 2007 [35]
EGFR and HER2 amplification	NO	YES	Missiaglia et al. 2014 [53]
CpG hypermethylation (CIMP)	YES	NO	Ogino et al. 2008 [45]
BRAF mutation (BRAF-like)	YES	NO	Popovici et al. 2012 [43]
KRAS	YES	NO	Slattery et al. 2009 [49]
p53	NO	YES	Klump et al. 2004 [54]
HOX gene	YES	NO	Sanz-Pamplona et al. 2011 [46]
CDX2 gene	YES	NO	Rozek et al. 2005 [48]
Thymidylate synthase	YES	NO	Edler et al. 2000a [65]; Liersch et al. 2006 [66]
Cyclin D3 and c-Myc	YES	NO	Slattery et al. 2009 [49]
Cyclin D1, cyclin E and nuclear β-catenin	NO	YES	Slattery et al. 2009 [49]
Activation of MAPK pathways	YES	NO	Iacopetta 2002 [50]
Activation of Wnt pathways	NO	YES	Peltomaki + Vasen 1997 [40]
Mucosal lesions (non-depressed type)	YES	NO	Konishi et al. 1999 [28]
Submucosal lesions (non-depressed type)	NO	YES	Konishi et al. 1999 [28]
Mucosal and submucosal lesions (depressed type)	YES	NO	Konishi et al. 1999 [28]

YES—Often positive or frequent incidence; NO—Often negative or rare incidence.

**Table 3:** Effects of different prevention measures on the two cancer entities.

Prevention Measure	Decreased Incidence	
	Colon Cancer	Rectal Cancer
Physical activity	YES (Halle and Schoenberg 2009 [75]; Slattery et al. 1997 [81]; Macfarlane and Lowenfels 1994 [74]; Chao et al. 2004 [79]; Larsson et al. 2007 [80]; Gerhardsson de Verdier et al. 1990 [78])	NO (Halle and Schoenberg 2009 [75]; Larsson et al. 2007 [80]; Chao et al. 2004 [79]; Gerhardsson de Verdier et al. 1990 [78])
Low BMI	YES (Slattery et al. 1997 [81]; Friedenreich et al. 2006 [82]; Larsson et al. 2007 [80])	NO (Friedenreich et al. 2006 [82]; Larsson et al. 2007 [80])
Reduced energy uptake	YES (Slattery et al. 1997 [81])	NO (Friedenreich et al. 2006 [82])
COX-2 inhibitors	NO in case of HNPCC (there are no sufficient data)	YES in case of FAP (Steinbach et al. 2000 [83]; Higuchi et al. 2003 [84]; Almendingen et al. 2010 [85])
Aspirin	YES (Rothwell et al. 2010 [86])	NO (Rothwell et al. 2010 [86])

HNPCC—Hereditary non-polyposis colorectal cancer; FAP—familial adenomatous polyposis coli.

**Table 4:** Comparison of long-term results for RC after laparoscopic versus open rectal resection (Ptok et al. 2006 [104]).

Header	Laparoscopic Resections	Converted Resections	Open Resections	*p*
FU rate [*n*]	150/192	27/33	4611/5782	–
Follow-up [months], mean (SD)	34 (12.3)	37 (12.7)	33 (13.4)	0.208
Local recurrences [*n*] ([%])	3 (2.0)	3 (11.1)	314 (6.8)	–
Metastasis [*n*] ([%])	18 (12.0)	4 (14.8)	553 (11.9)	–
Disease-free survival time (DFS) [months], mean (95% CI)	58.3 (55.3–61.3)	55.3 (47.2–63.4)	59.6 (58.8–60.5)	0.585
5-year survival rate [%]	83.8% (0.036)	73.4% (0.096)	74.5 (0.018)	
UICC stage-adapted				0.797

CI—Confidence interval, SD—standard deviation, *n*—number of patients, *p*—significance value.

**Table 5:** Comparison of the seven-year survival rates after adjuvant chemotherapy of CC (FOGT-1) [1] and radiochemotherapy for RC (FOGT-2) [2] (modified according to Kornmann et al. 2013 [109]).

Tumor Type	Colon Cancer		Rectum Cancer	
	N	7-Year Survival Rates (95% CI)	N	7-Year Survival Rates (95% CI)
**Treatment**				
5-FU	282	54.1% (46.5–61.0)	282	50.6% (43.0–57.7)
5-FU + FA	295	66.8% (59.4–73.1)	291	56.3% (49.4–62.7)
5-FU + IFN-α	278	56.7% (49.3–63.4)	223	54.8% (46.7–62.2)
**Treatment—UICC stage II**				
5-FU	21	61.9% (38.1–78.8)	93	57.5% (43.4–69.3)
5-FU + FA	23	78.3% (55.4–90.3)	97	75.9% (62.8–85.0)
5-FU + IFN-α	24	69.6% (46.6–84.2)	81	69.3% (55.0–79.9)
**Treatment—UICC stage III**				
5-FU	261	52.8% (44.6–60.4)	189	47.7% (38.8–56.2)
5-FU + FA	272	65.6% (57.7–72.3)	194	46.6%(38.6–54.2)
5-FU + IFN-α	254	55.3% (47.4–62.4)	142	46.1% (36.0–55.5)
**UICC substages**				
II (pT3–4pN0) *	68 *	70.1% (57.5–79.5)	271 *	67.2% (59.4–73.9)
IIIa (pT1–2pN1)	72	79.5% (62.4–89.4)	71	61.5% (47.9–72.4)
IIIb (pT3–4pN1)	424	62.2% (55.7–68.0)	227	52.7% (44.7–60.0)
IIIc (pT1–4pN2)	291	46.4% (39.4–53.2)	227	35.7% (26.9–44.5)
**Tumor grade**				
1–2	601	60.4% (55.2–65.2)	605	56.8% (51.8–61.5)
3	215	57.2% (48.5–64.9)	158	43.1% (34.3–51.6)
**Type of resection**				
Colon (all)	855	59.2% (54.9–63.2)	–	–
AR	–	–	359	57.0% (50.5–62.9)
APRE	–	–	188	45.3% (36.9–53.4)
Unknown	–	–	249	57.6% (50.5–64.0)

AR—Anterior resection, APRE—abdominoperineal rectal extirpation, 5-FU—5-fluorouracil, FA—folic acid, IFN—interferon, OS—overall survival, CI—confidence interval, UICC—Union for International Cancer Control; * T4 was excluded only by way of exception, as the patients had been treated with RCT at the start of the study.

**Table 6:** Local recurrence rate after curative resection without multimodal therapy in colon and rectal cancer adapted from [110].

Tumor Location	UICC Stage	Local Recurrence Rate (Only)	Combined Local Metastases Including Distant Metastases
Colon	I	7–8% ^a^	7–8% ^a^
	II	0–4% ^a^	14–43% ^a^
	III	0–7% ^a^	22–67% ^a^
Rectum	I	6–17% ^b^	12–18% ^b^
	II	13–24% ^b^	32% ^b^
	III	3–50% ^b^	37–64% ^b^

^a^ Bethune 1987 [111]; ^b^ Minsky et al. 1988 [112].

**Table 7:** Frequency and location of recurrence after adjuvant chemo- or radiochemotherapy of CC (FOGT-1) and RC (FOGT-2) (modified according to Kornmann et al. 2013 [109]).

Tumor Type	Colon Cancer				Rectum Cancer			
Variability	FOGT-1, Treatment			N	FOGT-2, Treatment			N
	A	B	C		A	B	C	
Basic Treatment	5-FU	5-FU	5-FU		5-FU	5-FU	5-FU	
Additional Treatment	–	+FA	+IFNα		–	+FA	+IFNα	
Number of Patients	282	295	278	855	282	291	223	796
N	128	112	112	352	129	123	97	349
Total recurrence rate	45.5%	38.0%	40.3%	41.2%	45.7%	42.3%	43.5%	43.8%
Local recurrence (only)	10	14	13	37	21	16	18	55
	3.5%	4.7%	4.7%	4.2%	7.4%	5.5%	8.1%	6.9%
Local recurrence with distant metastases	16	10	9	35	18	15	12	45
	5.7%	3.4%	3.2%	4.1%	6.4%	5.2%	5.4%	5.7%
Distant metastasis rate (only)	99	82	88	269	88	88	63	239
	35.1%	27.8%	31.7%	31.5%	31.2%	30.2%	28.3%	30.0%
Recurrence n.s. for the location	3	6	2	11	2	4	4	10
	1.1%	2.0%	0.7%	1.3%	0.7%	1.4%	1.8%	1.3%
**Distant metastases ^a^**								
Liver	66	52	60	178	60	54	42	156
	23.4%	17.7%	21.6%	20.8%	21.2%	18.5%	18.8%	19.5%
Lung	29	17	16	62	41	35	25	101
	10.2%	5.7%	5.7%	7.25%	14.5%	12.0%	11.2%	12.6%
Peritoneum	28	21	27	76	16	8	8	32
	10%	7.1%	9.7%	8.9%	5.6%	2.7%	3.5%	4.0%
Bone	0	3	3	6	2	11	4	17
		1.0%	1.0%	0.7%	0.7%	0.4%	1.8%	2.1%
Other locations	29	24	26	79	23	22	17	62
	10.2%	8.1%	9.3%	9.2%	8.1%	7.6%	7.6%	7.8%

^a^ Number of distant metastases: As some patients had more than one distant metastasis, the number of distant metastases is higher than the number of patients. N—Total number of patients, n—Total number of patients with recurrence, FA—Folic acid, 5-FU—5-fluorouracil, IFNα – interferon α, n.s.—Not specified.

## Abbreviations

CC	Colon cancer
CT	Chemotherapy
RC	Rectal cancer
CRC	Colorectal cancer
FOGT	Research Group Oncology of Gastrointestinal Tumors
LGIN/HGIN	Low grade or high grade intraepithelial neoplasia
FAP	Familial adenomatous polyposis
HNPCC	Hereditary non-polyposis colorectal cancer
APC	Adenomatous polyposis coli
MSI	Microsatellite instability
RCT	Radiochemotherapy
